# Characterization of functionally deficient SIM2 variants found in patients with neurological phenotypes

**DOI:** 10.1042/BCJ20220209

**Published:** 2022-07-13

**Authors:** Emily L. Button, Joseph J. Rossi, Daniel P. McDougal, John B. Bruning, Daniel J. Peet, David C. Bersten, Jill A. Rosenfeld, Murray L. Whitelaw

**Affiliations:** 1 Department of Molecular and Biomedical Science, University of Adelaide, Adelaide, Australia; 2 Institute of Photonics and Advanced Sensing, School of Biological Sciences, University of Adelaide, Adelaide, Australia; 3 Department of Molecular and Human Genetics, Baylor College of Medicine, Houston, TX, U.S.A.; 4 Baylor Genetics Laboratories, Houston, TX, U.S.A.

**Keywords:** aryl hydrocarbon nuclear receptor translocator, mutation, single-nucleotide polymorphisms, single-minded 2, transcription factors

## Abstract

Single-minded 2 (SIM2) is a neuron-enriched basic Helix–Loop–Helix/PER–ARNT–SIM (bHLH/PAS) transcription factor essential for mammalian survival. *SIM2* is located within the Down syndrome critical region (DSCR) of chromosome 21, and manipulation in mouse models suggests *Sim2* may play a role in brain development and function. During the screening of a clinical exome sequencing database, nine *SIM2* non-synonymous mutations were found which were subsequently investigated for impaired function using cell-based reporter gene assays. Many of these human variants attenuated abilities to activate transcription and were further characterized to determine the mechanisms underpinning their deficiencies. These included impaired partner protein dimerization, reduced DNA binding, and reduced expression and nuclear localization. This study highlighted several *SIM2* variants found in patients with disabilities and validated a candidate set as potentially contributing to pathology.

## Introduction

**Figure 1.**
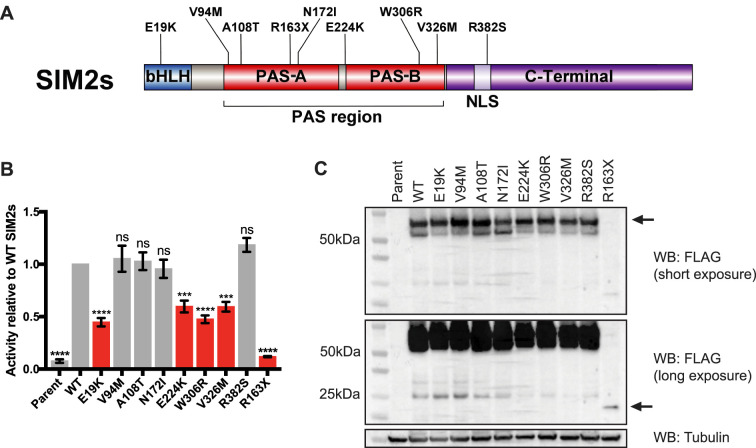
SIM2 variants have reduced activity on a CME reporter. (**A**) Schematic of SIM2s protein with domains and variants tested shown. N-terminal bHLH domain functions in DNA binding and primary dimerization. PAS domains are important for secondary dimerization and partner protein specificity. NLS directs SIM2 protein import into the nucleus. The C-terminal half of SIM2 contains transcriptional regulatory regions. Variants selected for functional testing are displayed above. (**B**) Dual-luciferase assays with a 6xCME reporter gene. Expression of SIM2s-HF and variants was induced with dox. Variants highlighted in red showed a significant reduction in reporter activity compared with WT SIM2s. Graph represents mean of *n* = 3 independent experiments, normalized to WT SIM2s control. Error bars represent SEM. Statistical significance determined by one-way ANOVA. *** *P *≤ 0.001, **** *P *≤ 0.0001, ns, not significant. (**C**) Western blot from whole-cell extracts. SIM2s-HF and variants were detected with FLAG antibody. Arrow indicates SIM2s variants, with the truncated *R163X* variant only detectable on long exposure.

Single-minded 2 (SIM2) is a member of the basic Helix–Loop–Helix/PER–ARNT–SIM (bHLH/PAS) family of transcription factors, which are broadly known to play important roles in development, homeostasis and cellular stress responses. These factors dimerize with a general bHLH/PAS partner protein (e.g. aryl hydrocarbon nuclear receptor translocator (ARNT) or the neuronally enriched paralog, ARNT2) to become DNA binding, functional transcription factors. This protein family is characterized by their N-terminal bHLH domain, required for DNA binding and primary dimerization, followed by a PAS domain consisting of two repeats, PAS-A and PAS-B, which function as secondary dimerization interfaces and confer partner protein specificity. Their C-terminal halves contain transcription regulatory domains (Figure 1A) [1–3]. SIM2/ARNT2 heterodimers bind to the central midline element (CME) DNA binding site to regulate gene expression [4,5]. Two isoforms of SIM2, SIM2-long (SIM2l) and SIM2-short (SIM2s), arise due to alternative splicing of the *SIM2* gene, with each isoform containing a unique C-terminus [6,7]. Two transrepression domains are located in the C-terminal half of the long isoform, with the short isoform only containing one. Both SIM2l and SIM2s have shown the ability to activate or repress transcription, dependent on context [5,7–10]. Target genes and cofactors associated with SIM2 are still largely unknown.

In mice, *Sim2* mRNA is expressed within the brain both during embryonic development and postnatally, indicating that *Sim2* may have important neural functions [4,11,12]. The location of *SIM2* within the Down syndrome critical region (DSCR) on human chromosome 21 stimulated the proposal that *SIM2* may be one of the genes contributing to the complex aetiology of this condition [6,13]. Supporting this notion, studies characterizing phenotypes of transgenic mice overexpressing *Sim2* reported neuronal/behavioural anomalies of reduced sensitivity to pain and anxiety-related/reduced exploratory behaviour [14,15]. *Sim2* knockout mouse models revealed *Sim2* to be an essential gene, with null mice dying perinatally. Interestingly, newborn *Sim2* knockout pups display many structural defects, including facial dysmorphologies such as cleft palates and skeletal defects of incompletely penetrant congenital scoliosis and abnormal rib protrusions [16,17]*. Sim2* KO mice also have impaired development of somatostatin and thyrotropin-releasing hormone-expressing neurons within the hypothalamus, leading to a reduction in the number of these neurons [18]. The mechanisms behind these phenotypes are yet to be elucidated, so the exact functions that *Sim2* plays during normal development are unclear. Overall, *Sim2* mouse models have shown that aberrant expression of *Sim2* can deleteriously affect brain development and/or function.

Given the phenotypes of *Sim2* KO mice, it is possible that mutations in *SIM2* may be causing or contributing to human developmental disorders including intellectual disabilities, facial dysmorphologies and congenital scoliosis. Exome sequencing data from patients with intellectual disabilities revealed many non-synonymous single-nucleotide variants in the *SIM2* gene. In this study, we functionally tested and characterized *SIM2* variants in order to determine whether they are candidates for causing or contributing to disorders in the patients harbouring those variants. Many patient variants showed a significant reduction in the ability to activate transcription of a reporter gene. These variants were further characterized to determine the mechanism behind the deficiency in protein activity. This work has highlighted many *SIM2* variants as candidates for disability causing or contributing mutations and expanded the set of amino acids that are essential for SIM2 to maintain complete function as a transcription factor.

## Results

### SIM2 variants in patients with neurological phenotypes

Clinical exome sequencing data were screened to look for rare variants in the *SIM2* gene. Many heterozygous, non-synonymous variants were found, and nine were selected for functional analysis based on their predicted effect on SIM2 protein function (Figure 1A, Table 1, Supplementary Table S1). All variants selected are either not present or present at low frequencies in the Genome Aggregation Database (gnomAD) database (v2.1.1) [19]. Except for the *E19K* variant, which was apparently mosaic in the proband and absent from the parents by trio exome sequencing, the variants were apparently heterozygous and found via proband exome sequencing, so the inheritance of the variants is unknown. The *E19K* variant is within the first helix of the bHLH domain and thus might disrupt DNA binding or dimerization with ARNT2. The *V94M*, *A108T* and *N172I* variants are within the PAS-A repeat of the PAS domain. *E224K*, found in three patients from two families, lies at the N-terminal border of the PAS-B repeat. *W306R* and *V326M* variants lie near the C-terminal end of the PAS-B repeat, positioned in a region known to be important for activity [20]. As the PAS domain is crucial for dimerization with ARNT2, all of these variants were logical choices for activity screening. Additionally, these variants are predicted to be probably damaging to the protein using PolyPhen-2 (Polymorphism Phenotyping v2) predictive software [21]. The *R382S* variant is within the nuclear localization signal (NLS) in the C-terminus of the protein. The *R163X* mutation introduces a premature stop codon truncating the protein in the PAS-A repeat which, therefore, is predicted to disrupt dimerization (Figure 1A). All variants are in protein-coding regions common to both the short and long isoform of SIM2. Phenotypes in common between some patients harbouring functionally deficient *SIM2* alleles include intellectual disabilities, delayed speech, seizure disorders, hypotonia, dysmorphic features and scoliosis. It is worth noting that some of these patients also have mutations in other genes that may well contribute, albeit to unknown degrees, to their complex phenotypes (see Table 1, Supplementary Table S1). Expression cassettes for the short isoform of SIM2 (SIM2s) and each variant, incorporating a 2xHA-3xFLAG epitope tag (SIM2s-HF), were integrated into a set position in the genome of the T-REx293 cell line. This provided a set of stable cell lines with doxycycline-inducible expression for WT SIM2s-HF and each variant for functional characterization of each protein.

**Table 1.** *SIM2* (NM_005069.6) gene variants found by clinical exome sequencing shown to be functionally deficient.

Sex	Age (years)	Phenotype	Variants thought to explain phenotypes	**Nucleotide (amino acid)** *Polyphen-2*	Reads	gnomAD Database (allele frequency)
F	19.4	Delayed motor milestones and speech, intellectual disability, hypotonia, seizure disorder, dysmorphic features, short stature, microcephaly, joint contractures, failure to thrive, cerebral palsy, scoliosis and sensitive skin.	Heterozygous c.116C>G (p.S39X) pathogenic variant in the SMC1A gene. SMC1A variants cause Cornelia de Lange syndrome 2 (CDLS2), an X-linked developmental disorder. Phenotypes include facial dysmorphisms, abnormal hands and feet, and growth and developmental delay [45].	**c.55G>A** **(p.E19K)** *probably* *damaging*	31/268	Not present
M	3.6	Dysmorphic features, reduced vision, mild hypotonia. Parents are consanguineous. Sibling of below patient.	Homozygous c.1340T>C (p.V447A) variant of unknown clinical significance (VUS) in the TUBGCP6. TUBGCP6 variants cause microcephaly and chorioretinopathy, autosomal recessive, 1 (MCCRP1). Phenotypes include microcephaly, cognitive and visual impairment [46].	**c.670G>A** **(p.E224K)** *probably* *damaging*	30/51	0.000007964
F	2.8	Dysmorphic features, reduced vision, unilateral reduced hearing. Parents are consanguineous. Sibling of above patient	Homozygous c.288T>A (p.C96X) pathogenic variant in the SPATA7 gene. SPATA7 variants cause Leber congenital amaurosis 3 (LCA3) and retinitis pigmentosa vision impairment disorders [47].	**c.670G>A** **(p.E224K)** *probably* *damaging*	26/47	
F	0.4	Elevated 3-methylglutaconic acid, hyperammonemia, and hypoglycaemia. Chromosomal microarray revealed large regions of absence of heterozygosity (AOH). Parents are consanguineous.	Homozygous c.442C>T (p.R148X) disease causing variant in the SERAC1 gene. SERAC1 variants cause 3-methylglutaconic aciduria with deafness, encephalopathy, and Leigh-like syndrome (MEGDEL), an autosomal recessive disorder. Phenotypes include delayed psychomotor development, hearing loss, movement disorder, and elevated 3-methylglutaconic acid [48].	**c.670G>A** **(p.E224K)** *probably* *damaging*	26/70	
M	8.8	Speech delay, autism, intellectual disability, spasticity, seizures, and joint contractures.		**c.916T>C** **(p.W306R)** *probably* *damaging*	17/37	0.000008107
F	17.5	Global developmental delay, failure to thrive, seizure disorders, hypertonia, nystagmus, microcephaly, mild scoliosis, decreased volume of grey and white matters and thinned corpus callosum of the brain.		**c.976G>A** **(p.V326M)** *probably* *damaging*	21/54	0.00002689
F	21.5	Autism, intellectual disability, hypotonia, hyperextensibility, tachycardia, cardiomyopathy, scoliosis, dysautonomia, and temperature intolerance.	Heterozygous c.490C>T (p.R164X) pathogenic variant in the HDAC8 gene previously reported to cause Cornelia de Lange syndrome 5 (CDLS5) (Deardorff et al 2012), an X-linked dominant developmental disorder. Phenotypes include facial dysmorphisms, abnormal hands and feet, and growth and developmental delay [49].	**c.487C>T** **(p.R163X)**	31/52	0.00003186

### SIM2 variants show reduced transcriptional activity on a reporter gene

To test whether the amino acid variants change the activity of SIM2s as a transcription factor, reporter gene assays were performed using the T-REx293 stable cell lines with induced expression of WT SIM2s or patient-derived variants. SIM2s was previously shown to activate the expression of a CME-based reporter gene *in vitro* [5,20,22]. There was no significant change in the activity of the *V94M*, *A108T*, *N172I* and *R382S* variants compared with WT SIM2s, suggesting that these amino acids are not critical for SIM2s function as a transcription factor in this system. These variants exhibit comparable potencies to WT SIM2s so are unlikely to be causing or contributing to any human phenotypes, thus were not analyzed any further. The *E19K*, *E224K*, *W306R* and *V326M* variants all showed approximately 50% reduction in reporter gene activity when compared with the WT protein, whereas the *R163X* variant had near null activity (Figure 1B). Western blot showed that WT SIM2s-HAFLAG and the variants were all expressed as expected in this system after dox induction; however, the *R163X* variant exhibited a low level of protein expression compared with the WT protein (Figure 1C). This may be because the truncated protein is less stable than the WT protein.

### Select SIM2 variants show reduced dimerization with partner protein ARNT2

**Figure 2.**
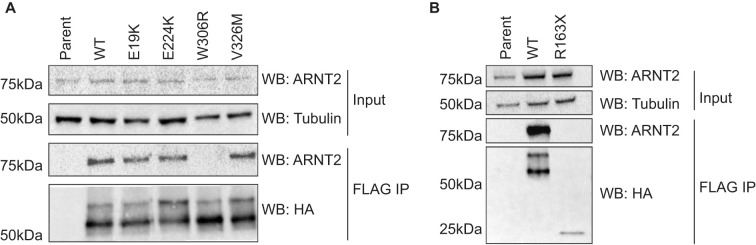
Select SIM2s variants show impaired dimerization with ARNT2. Co-immunoprecipitation experiments performed to determine the ability of SIM2s-HF and variants to dimerize with ARNT2. FLAG immunoprecipitation followed by western blots for ARNT2, HA (SIM2s-HF) and Tubulin were performed. Blots are representative of 3 independent experiments. (**A**) Expression of SIM2s-HF and the *E19K*, *E224K*, *W306R* and *V326M* variants induced by dox. (**B**) SIM2s-HF and *R163X* variants expressed at a 1 : 5 ratio.

To function as a transcription factor, SIM2 forms a heterodimer with ARNT2. Co-immunoprecipitation experiments were, therefore, performed to assess whether the activity-deficient variants had impaired dimerization with ARNT2. The *E19K*, *E224K* and *V326M* protein variants all appeared to co-immunoprecipitate ARNT2 to a similar extent to WT SIM2s, indicating that they are able to efficiently dimerize with ARNT2 (Figure 2A). In contrast, the *W306R* and *R163X* variants revealed weakened abilities to co-immunoprecipitate ARNT2 (Figure 2A,B). Due to the low level of expression of the R163X variant in the stable cell line, co-immunoprecipitations were repeated using extracts from 293T cells transiently transfected with WT or *R163X* expression plasmids (the latter in 5-fold excess to achieve comparable expression levels). Despite clear immunoprecipitation of *R163X*, a reproducible lack of ARNT2 co-immunoprecipitation was observed (Figure 2B). The weaker dimerization of *W306R* and *R163X* with ARNT2 is consistent with the decrease in activating potency of this variant, while for *R163X*, the weak dimerization with confounding low expression culminates in little or no activity (Figure 1B).

### Dimerization-deficient SIM2 variants show reduced competition with HIF1α

**Figure 3.**
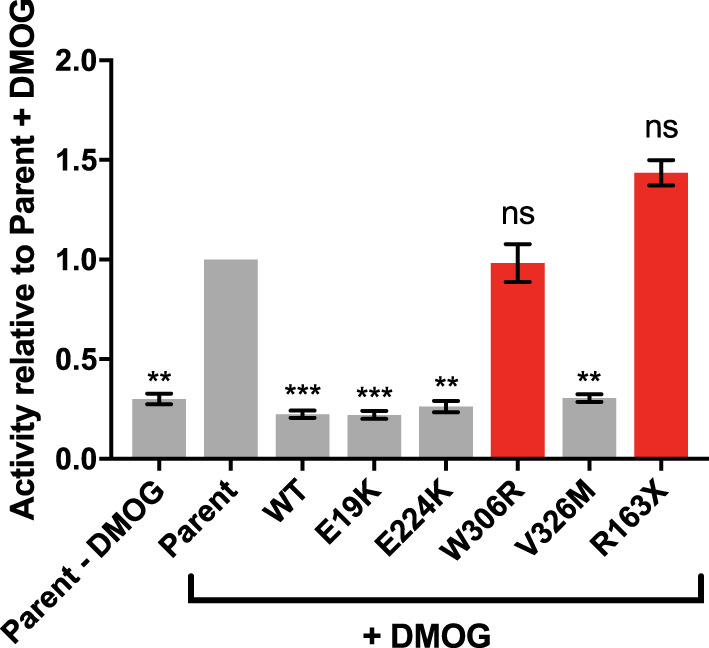
Dimerization-deficient variants lose the ability to repress HIF1α transcriptional activity. Dual-luciferase reporter assay with a HIF1α responsive 4xHRE reporter. HIF1α expression was induced with the hypoxia mimetic DMOG; expression of SIM2s-HF and variants were induced with dox. Variants highlighted in red show no repression of HIF1α-mediated activation of the reporter. Graph represents mean of *n* = 4 independent experiments, normalized to parent control. Error bars represent SEM. Statistical significance determined by one-way ANOVA. ** *P *≤ 0.01, *** *P *≤ 0.001, ns not significant.

It has been shown previously that SIM2 is able to repress HIF1α reporter gene activity through competitive binding with their common partner factors ARNT/ARNT2 [10,20]. To investigate if any of the SIM2 variants had a reduced ability to act as a transcription repressor in this way, we assayed a reporter gene driven by the HIF1α activated hypoxia response element (HRE) (Figure 3). When HIF1α expression was induced with the hypoxia mimetic dimethyloxalylglycine (DMOG), the activity of the reporter was increased as expected, confirming that HIF1α was dimerizing with ARNT/ARNT2 to form a functional transcription factor. When WT SIM2s expression was concomitantly induced with dox, the activity of the reporter was reduced to basal levels, indicating that SIM2s was competitively dimerizing with ARNT/ARNT2 to prevent HIF1α induction of the reporter. Agreeing with the immunoprecipitation data (Figure 2A,B), the *E19K*, *E224K* and *V326M* variants were all able to repress the HRE reporter gene to a similar extent as WT SIM2s. In contrast, the *W306R* and *R163X* variants were not able to repress HIF1α mediated reporter activity, consistent with attenuated SIM2s dimerization with ARNT/ARNT2.

### SIM2 variants maintain nuclear localization

**Figure 4.**
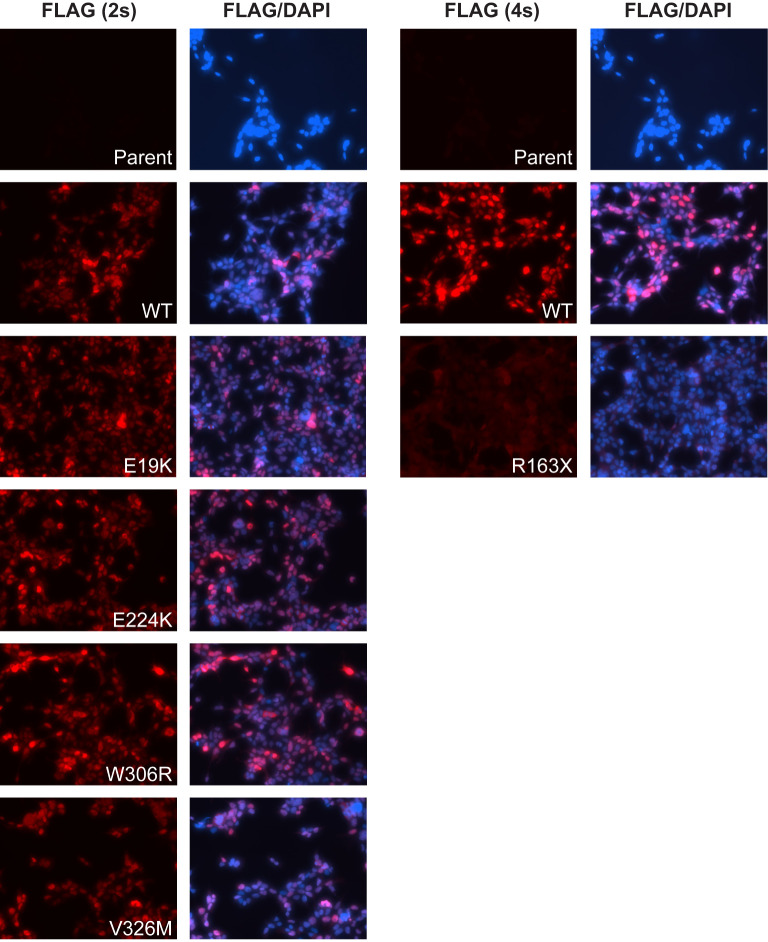
Cellular localization of SIM2 variants. Immunofluorescence of fixed T-REx293 cells dox induced to express WT SIM2s-HF or mutant variants. FLAG antibody used to detect SIM2 and variants shows all variants are localized to the nucleus except the *R163X* variant, which is localized throughout the entire cell. 2s; 2 second exposure, 4s; 4 second exposure.

SIM2 is localized to the nucleus of cells, and any change of this localization may dampen the transcriptional outputs of SIM2 [20,22,23]. Immunofluorescence was performed to determine whether any of the variants disrupted the transport of SIM2s to the nucleus. As shown in Figure 4, WT SIM2s is predominantly nuclear in T-REx293 cells. The *E19K*, *E224K*, *W306R* and *V326M* variants all show predominant nuclear localization, comparable to WT SIM2s. Therefore, altered cellular localization would not be affecting the activity of these variants. The *R163X* variant is expressed at a much lower level compared with WT SIM2s and also has a distinctly different localization, being distributed throughout the entire cell. This is not unexpected, as this variant is truncated before the NLS. This change in localization of *R163X*, additional to poor expression and minimal dimerization with ARNT2, explains the dramatically decreased activity when compared with the other attenuated SIM2s variants.

### DNA binding deficiency of the SIM2 E19K variant

**Figure 5.**
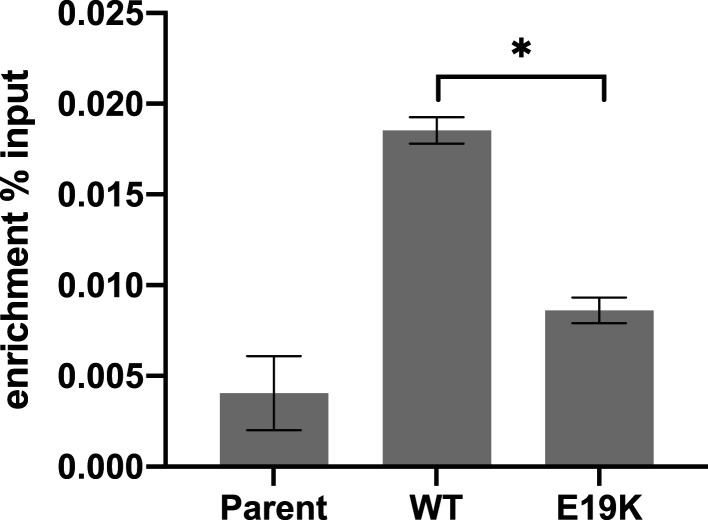
DNA binding of the *E19K* variant. SIM2 Chromatin immunoprecipitation assays from cells with dox-induced expression of SIM2s-HF or *E19K* variant. Enrichment of the 6xCME response element was assessed by qPCR. Graph represents mean of *n* = 3 independent experiments presented as percent enrichment compared with input. Error bars represent SD. Statistical significance determined by one-way ANOVA. * *P *≤ 0.05 *** *P *≤ 0.001.

*E19K* is the sole variant located in the basic DNA binding region of the protein, highlighting this residue as potentially involved in SIM2s/ARNT2 dimer interaction with the DNA response element, CME. To investigate this, chromatin immunoprecipitation (ChIP) experiments were performed by inducing expression of SIM2s WT or the *E19K* variant and assessing enrichment of the 6xCME response element in the pML-6xCME reporter plasmid. Significant enrichment of the 6xCME response element was observed in the WT SIM2s cell line compared with the parent cell line (Figure 5), demonstrating that SIM2s directly binds to the 6xCME response element in the reporter plasmid to activate the expression of the reporter gene. Compared with the WT protein, the *E19K* variant showed approximately a 50% reduction in the enrichment of the 6xCME DNA binding region. Given that *E19K* is not deleterious for nuclear localization (Figure 4) or dimerization with ARNT2 (Figures 2A and 3), reduced DNA binding capability is the most likely cause for decreased transcriptional activity observed for this variant.

### Modelling of the SIM2 : ARNT2 : DNA heterodimer structure

**Figure 6.**
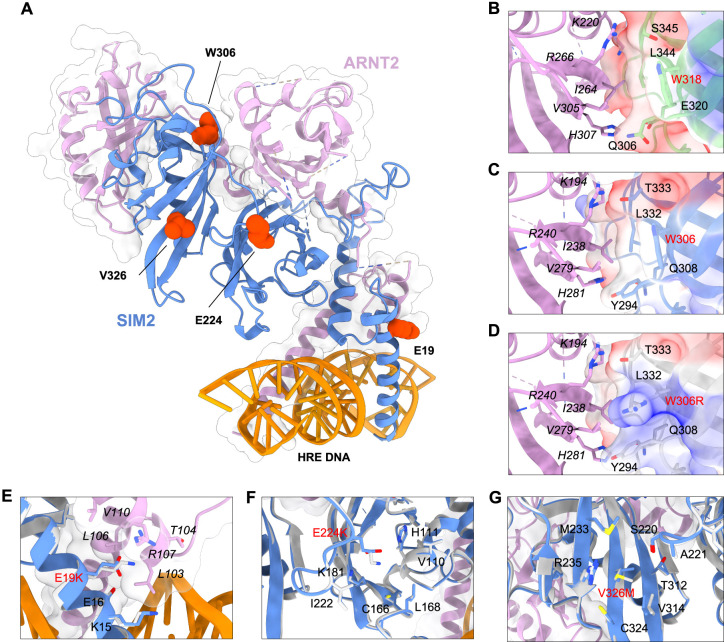
SIM2 : ARNT2 : DNA structural models. (**A**) Homology model of the WT SIM2 : ARNT2 : DNA structure based on the mouse HIF2α : ARNT : DNA co-crystal structure (PDB:4ZPK). SIM2 is depicted in blue, ARNT2 in purple with transparent surface shown to highlight protein–protein interfaces. SIM2 missense variant residues are shown as red spheres. (**B**) PAS-A/PAS-B interface of the mouse HIF2α : ARNT : DNA co-crystal structure (PDB:4ZPK). Interface residues are shown as sticks and clearly labelled. Surface electrostatics of the HIF2α are shown. (**C**) PAS-A/PAS-B interface of the WT SIM2 : ARNT2 model and (**D**) mutant W306R SIM2 : ARNT2 model. Surface electrostatics are shown to demonstrate charge disruption introduced by W306R mutation. (**E–G**) Mutations E19K, E224K and V326M, respectively. ARNT1/2 residue labels are represented in italics for clear differentiation, and mutant residues are shown in red.

Given that no X-ray crystal structure is available for the SIM2 : ARNT2 : DNA complex, a homology model was created to better understand the atomic details regarding the variants analyzed in this study. The model was based on the mouse HIF2α : ARNT : DNA bHLH/PAS-A/PAS-B co-crystal structure (PDB 4ZPK), as HIF2α is the most closely related bHLH/PAS factor to SIM2 with a solved crystal structure [24]. We created a wildtype model that closely mimicked chain lengths, and also modelled mutations for *E19K*, *E224K*, *W306R* and *V326M* (Figure 6A). All mutations except *V326M* change the charge of the amino acid, indicating that these substitutions may alter the charge state within the local vicinity of the position, likely interfering with intramolecular interactions and/or the interaction energy between subunits of the dimer. The Trp to Arg mutation of SIM2 at position 306 is found at the SIM2 : ARNT2 dimer interface between the PAS-A and PAS-B domains, at an identical position to HIF2α *Trp318* in the HIF2α : ARNT crystal structure (Figure 6B,C). Within the SIM2 PAS-B domain, *Trp306* likely makes hydrophobic interactions (and potentially pi–pi stacking in a dynamic environment) with *Tyr294* and also with *Leu332*, which itself makes hydrophobic interactions with *Tyr294.* Together, these residuesform a small hydrophobic cluster. This cluster is likely to form favourable inter-domain hydrophobic interactions *Ile238* and *Val279* of ARNT2 PAS-A. Mutation of *Trp306* to Arg would disrupt interdomain hydrophobic interactions and likely perturb interactions between SIM2 *Tyr294* and *Leu332* by (1) altering charge at the interface and (2) sterically affecting interface conformation (Figure 6C,D). The *E19K* mutation disrupts charge complementarity at the dimer interface close to the DNA binding surface, abrogating a potential salt bridge between *Glu19* of SIM2 and *Arg107* of ARNT2 and destabilization of helical secondary structure (Figure 6E). This provides an explanation for the observed weakened affinity for DNA and deficient activity. Mutation *E224K* located on the PAS-A/B loop also disrupts the charge adjacent to the interface (Figure 6F). However, it is difficult to make accurate predictions regarding the effect of *E224K*, as like the crystal structure, the model is missing many residues from the ARNT2 PAS-A domain. The *V326M* mutation is found on the PAS-B β-sheet directly opposing the PAS-A/B loop. The much larger side chain of Met at position 326 of SIM2 likely causes a steric clash with the tip of the loop containing *Ser220* of SIM2, possibly reorganizing the loop at the SIM2:ARNT2 interface and disrupting allostery between the two domains (Figure 6F).

## Discussion

Although *SIM2* is an essential gene, its exact functions and molecular pathways are still not well understood. Mouse models have established that deletion of the *Sim2* gene produces a variety of developmental phenotypes; however, the extent to which *SIM2* gene variants cause or contribute to human pathologies remains undetermined. Recently a study proposed a homozygous SIM2 variant (p.Tyr154Cys) as the cause of the clinical presentation in a child with craniofacial abnormalities, developmental delay and intellectual disability [25]. Understanding the molecular basis of how non-synonymous nucleotide variants can alter the activity of SIM2 will not only provide a greater understanding of the way SIM2 functions as a transcription factor but could also highlight the possibility of these variants causing or contributing to human disease.

To study whether single amino acid SIM2 variants found in patients with intellectual disabilities cause functional changes in activity, we used an *in vitro* cell-based system. This consisted of stable cell lines harbouring site-specific inducible expression cassettes for WT SIM2s or mutant variants, which were used to compare relative outputs on a reporter gene. Out of the nine variants selected from the patient exome sequencing dataset, five of these showed a clear significant reduction in transcriptional activity compared with the WT protein (Figure 1). These variants lie across known important functional domains of the protein where there is strong potential for amino acid changes to disrupt protein function. While there are no known human variants linked with human disease as of yet, this finding highlights the possibility that human SIM2 variants can significantly change protein activity.

Further functional analysis of the activity-deficient variants was performed in order to elucidate the mechanism behind the reduced transcriptional activity. As SIM2 must dimerize with ARNT or ARNT2 to form a functional transcription factor, co-immunoprecipitation experiments were used to determine the capacity of these variants to form dimers with ARNT2. This showed that the *W306R* and *R163X* variants had little or no capacity to dimerize with ARNT2. The *W306R* variant lies within the previously defined ‘hot spot' region for dimerization [20], confirming that residues within this region form a critical interaction surface. The *R163X* variant is truncated after the first PAS repeat of the PAS domain (PAS-A). It has been shown previously that the entire PAS domain (PAS-A and PAS-B) is important for SIM2 to dimerize with ARNT2 [20]. Therefore, our truncated variant lacking PAS-B confirmed the expected dimerization deficiency. Consistent with previously reported data for the closely related protein SIM1, where the variant SIM1 *V326F* did not influence dimerization [20], the identically located SIM2s *V326M* variant had no change in ARNT2 dimerization compared with the WT protein.

Complementary to the co-immunoprecipitation data, competition reporter gene assays showed that the *W306R* and *R163X* variants failed to repress the activity of the related bHLH/PAS factor, HIF1α, whereas WT SIM2 and the dimerization efficient variants afforded near full repression of HIF1α on a hypoxic response element. While the functional relevance of the competition between HIF1α and SIM2 *in vivo* during development is yet to be established, this loss of competition could have functional and phenotypic consequences. The interaction between genes and the environment has been implicated in the penetrance of scoliosis. Instances of hypoxia during embryogenesis can significantly increase the penetrance of scoliosis in mice that are heterozygous for genes associated with human scoliosis [26]. Many the patients with *SIM2* variants (*E19K*, *V326M*, *R163X*) have scoliosis, a phenotype that is seen in both the heterozygous and homozygous *Sim2* knockout mouse model with incomplete penetrance. Together these observations suggest that *SIM2* may be a susceptibility allele for scoliosis, increasing the incidence of this phenotype, likely in combination with environmental stresses (such as hypoxia) or other gene variants. It is possible that the competition between SIM2 and HIF1α may come into play here, which could be tested through generating mouse models of these variants and observing the penetrance of scoliosis with and without other environmental stresses, such as hypoxic events during embryogenesis, or genetic variants which have been shown to promote scoliosis [26].

It has previously been established that SIM2 is localized to the nucleus in cells [20,23]. Immunofluorescence analysis of the SIM2 variants showed that most are localized to the nucleus, with the exception of the *R163X*, which is localized throughout the cell. As this variant is truncated before the NLS and small in size, this result is to be expected. While such a change in localization would dampen transcriptional outputs of SIM2, we found this variant was also defective for ARNT2 dimerization and additionally has a lower level of expression compared with the WT protein. These properties in combination are the likely reason why this variant has essentially null activity on the CME reporter gene. It is also possible that the native *R163X* transcript would be subject to nonsense-mediated decay *in vivo* due to the introduction of a premature stop codon, resulting in severely reduced protein being produced from this allele. As the truncated protein is being expressed from cDNA in our experimental cell lines, it was not possible for us to test if this is the case.

Homology modelling indicated that the *E19K* and *W306R* activity-deficient missense variants are likely located at SIM2:ARNT2 dimer interfaces, while *E224* and *V326M* lie adjacent to interfaces. However, by co-immunoprecipitation only one (*W306R*) showed a clear reduction in dimerization, suggesting that structural perturbations occurring at or near interfaces affect other protein properties important for activity, such as allostery. Analysis of the mouse HIF-2α : ARNT crystal structure (4ZPK) suggests a conserved role for HIF-2α *W318* (equivalent position to *W306* in SIM2) in promoting favourable interaction with the ARNT PAS-A domain (Figure 6B). Strikingly, crystal structures show this interaction is disrupted by the HIFa antagonist proflavine, which prevents HIFa/ARNT dimerization in biochemical assays [24,27]. Notably, Wu et al. [28] identified this Trp as being highly conserved in bHLH/PAS family members that dimerize with ARNT or ARNT2, suggesting its crucial role in dimerization that we have established for SIM2 may be general within the protein family. It is, therefore, also likely that mutation of Trp at equivalent positions in ARNT2 partner proteins would have deleterious effects on heterodimerization and ultimately function.

The *E19K* variant is close in proximity to the basic DNA binding region but does not appear to be in direct contact with the DNA response element. ChIP studies showed that the *E19K* variant has reduced DNA binding capabilities compared with the WT protein, so it is possible that this variant may be disrupting DNA binding through structural changes of the bHLH-motif caused by physicochemical properties of the E to K change. The *E224K* and *V326M* variants lie within regions encompassing the PAS-B domain, which is known to serve as a ligand-binding domain for other members of the bHLH/PAS protein family [29]. PAS domains are also known to mediate protein : protein interactions [30–34]. It is, therefore, possible that these variants are disrupting interactions with endogenous ligands or cofactors that are required for SIM2s function, or disrupt intramolecular allostery between the SIM2 PAS domains. As there are no known ligands for SIM2 and cofactors required for SIM2s transcriptional activity are yet to be discovered, these models cannot currently be tested.

While this study clearly shows that these SIM2 variants are transcriptionally deficient within the *in vitro* cell-based assays, these data do not show that these effects alone would translate to a clinical phenotype in patients. However, it is worth noting that while there were some commonalities in phenotypes for patients harbouring these variants, the complete clinical phenotypes were broadly diverse, and some patients also harbour gene variants that have either been linked to, or are potentially associated with, human pathologies (see Table 1). These observations suggest that rather than *SIM2* variant alleles being directly causative of human pathologies, it is more likely that the variants represent susceptibility alleles, where deficient SIM2 functions contribute to human pathologies in combination with other gene variants. In addition, the *SIM2* variants reported in this study when present in a homozygous or compound heterozygous state may result in developmental abnormalities in humans. Future animal studies that recapitulate the *SIM2* variants will be critical to assess their relative contribution(s) to the patient developmental phenotypes. In conclusion, this study has identified an additional set of four amino acids that are important for SIM2 to function as a transcription factor and provides a reference list that will be useful as more patients with intellectual disability and/or developmental abnormalities are discovered to harbour *SIM2* anomalies.

## Materials and methods

### Exome sequencing

A database of ∼8600 patients undergoing clinical exome sequencing at Baylor Genetics was searched for rare *SIM2* variants. A majority of patients (∼78%) were referred for phenotypes including neurologic symptoms. Most individuals (∼92%) had proband-only sequencing, so the inheritance of variants was not determined. Exome sequencing was conducted according to previously described methods [35].

### Plasmid construction

Human SIM2s-2xHA-3xFLAG and variants were generated through PCR amplification and mutagenesis and cloned into pENTR1A by Gibson assembly [36]. These were then subcloned into pcDNA5-FRT/TO-Gateway through LR recombination according to the manufacturer's instructions (Invitrogen). Plasmids pEF-ARNT2-IRES-neo [37] and pGL3-4HRE luciferase [38] have been described previously. pML-6CME was a gift from Dr J Pelletier (Department of Biochemistry, McGill University, Montreal, Canada).

### Generation and maintenance of cell lines

Cells were cultivated in DMEM (Dulbecco's modified Eagle's medium; Gibco) with 10% FBS (Gibco), 2 mM GlutaMAX (Gibco), 10 000 units/ml penicillin and 10 mg/ml Streptomycin (Invitrogen) at 37°C, 5% CO_2_. Stable cell lines were generated using the Flp-in T-REx293 system according to the manufacturer's instructions (Invitrogen).

### Dual-luciferase reporter assays

*CME SIM2 activity assays:* T-REx293 stable cell lines were seeded in a 24-well tray in triplicate. The following day cells were transfected with 0.1 ng pRL-CMV (Promega), 400 ng pML-6xCME and 50 ng of pEF-ARNT2-IRES-neo using PEI (polyethylenimine) (Polysciences, U.S.A.) transfection reagent [39]. Seven hours after transfection media was replaced with complete medium containing 1 µg/ml doxycycline (dox) (Sigma). Cells were harvested 20 h after dox induction with passive lysis buffer (Promega). Dual Luciferase assays (Promega) were carried out according to the manufacturer's protocol using Promega GloMax™ 96 Microplate Luminometer for at least three biological replicates. To calculate relative luciferase units (RLUs) Firefly luciferase units were normalized to Renilla luciferase units. RLUs were then used to calculate the means of the triplicate transfections for each biological replicate.

*HIF1α competition assay:* T-REx293 stable cell lines were seeded as above and transfected with 0.1 ng pCMV-RL (renilla luciferase) and 400 ng pGL3-4xHRE luciferase. Seven hours post-transfection media was replaced with complete medium containing 1 µg/ml dox and 1 mM DMOG (Sigma–Aldrich) to induce HIF1α expression. Twenty hours after dox and DMOG treatment cells were harvested and dual-luciferase assays were performed as above.

### Immunoblotting

Samples were separated by SDS–PAGE using 7.5%, 12% or 4–20% Mini-PROTEAN® TGX™ Precast Gels (Bio-Rad) before being transferred to nitrocellulose membranes using Trans-Blot® Turbo™ Transfer System (Bio-Rad) and probed with the following primary antibodies; anti-FLAG (M2, Sigma), anti-ARNT2 (M165, Santa Cruz Biotechnology), anti-HA (C29F4, Cell Signalling) and anti-α-tubulin (MCA78G, Bio-Rad). Blots were probed with the following secondary antibodies; goat anti-mouse HRP (Pierce), goat anti-rabbit HRP (Pierce) and rabbit anti-rat HRP (Dako) and developed using Clarity™ Western ECL Blotting Substrates (Bio-Rad). Blots were imaged with a Bio-Rad ChemiDoc MP Imaging System.

### Immunoprecipitation

For the SIM2s WT, *E19K*, *E224K*, *W306R* and *V326M* variant immunoprecipitations (IP); T-REx293 cells were treated with 1 µg/µl dox for 6 h before lysis and protein extraction. For the *R163X* variant IP; HEK 293T cells were transfected with 1 µg of pEF-ARNT2-IRES-neo and either 1 µg of pcDNA5-FRT/TO-SIM2s-HA-FLAG plasmid or 5 µg pcDNA5-FRT/TO-R163X-SIM2s-HA-FLAG plasmid. Proteins were immunoprecipitated with FLAG M2 resin (Sigma–Aldrich) overnight at 4°C. Following washes, proteins were eluted from the resin by boiling in 4× SDS load buffer (20% glycerol, 2.5% SDS, 200 mM DTT) and analyzed by immunoblotting.

### Immunocytochemistry

Cells were seeded on a glass coverslip and allowed to settle overnight. After treatment for 24 h with dox, cells were fixed with 4% PFA (Sigma), permeabilized with 0.2% Triton X-100 (Sigma) and blocked with 10% normal horse serum. Coverslips were incubated with anti-FLAG antibody (M2, Sigma) overnight at 4°C followed by donkey α-mouse Alexa Fluor® 594 (Invitrogen) at room temperature for 2 h. Coverslips were then mounted onto glass slides with ProLong Gold antifade reagent with DAPI (Invitrogen). Images were taken using a Nikon Eclipse Ti microscope and Nikon Digital Sight DS-Qi1 camera.

### Chromatin immunoprecipitation (ChIP)

Cells were seeded in a T175 flask and allowed to settle overnight before being transfected with 20 µg pML-6xCME using PEI transfection reagent. Seven hours after transfection media was replaced with complete medium containing 1 µg/ml doxycycline (dox) (Sigma). After 24 h dox induction cells were cross-linked with 1% formaldehyde for 10 min at room temperature. Chromatin extracts were prepared and immunoprecipitation performed as described previously [40]. Immunoprecipitation was performed with 5 µg of anti-SIM2 antibody (21069-1-AP, Proteintech), or control anti-rabbit antibody (cyclin D2, M-20, Santa Cruz Biotechnology). ChIP samples were purified through NucleoSpin DNA columns (Macherey-Nagel) using QIAGEN PCR Purification kit buffers according to the manufacturer's instructions and analyzed by qPCR using primers designed to amplify the 6xCME region of the pML-6xCME reporter plasmid.

### Statistics

Data from the dual-luciferase assays are presented normalized to the control of that group (WT SIM2s for CME activity assays or parent T-REx293 line + DMOG for HIF1α competition assays) as mean; error bars represent the standard error of the mean (±SEM). Data from the ChIP assays are presented as percent enrichment of the 6xCME response element compared with input; error bars represent standard deviation (±SD). Statistical analysis was performed on log-transformed data. One-way ANOVA followed by Dunnett's multiple comparisons test was performed using GraphPad Prism version 9.0 for MacOS, GraphPad Software, La Jolla California U.S.A., www.graphpad.com.

### Homology modelling

All simulations were carried out in ICM Pro software suite (Molsoft) [41]. Homology models were carried out in the Homology Model module of ICM Pro [42,43]. SIM2 (Uniprot: Q14190) and ARNT2 (Uniprot: Q9HBZ2) homology models were first created separately and docked together, and on DNA, using the HIF2α : ARNT : DNA co-crystal structure (PDB:4ZPK) as a reference [24]. Further regularization, minimization and model refinement were then carried out to ensure the integrity of the dimer interface and interactions of the individual mutations. Figures were created using UCSF ChimeraX [44].

## Data Availability

All data and reagents are available upon request.

## Open Access

Open access for this article was enabled by the participation of University of Adelaide in an all-inclusive *Read & Publish* agreement with Portland Press and the Biochemical Society under a transformative agreement with CAUL.

## Abbreviations

ChIP: chromatin immunoprecipitation
CME: central midline element
DSCR: Down syndrome critical region
HRE: hypoxia response element
IP: immunoprecipitations
NLS: nuclear localization signal
PEI: polyethylenimine
SIM2: single-minded 2
SIM2l: SIM2-long
SIM2s: SIM2-short
