# Supplementary material for: Characterization of functionally deficient SIM2 variants found in patients with neurological phenotypes
Source: Biochem J. 2022 Jul 13;479(13):1441–54. doi: 10.1042/BCJ20220209 (PMC9342896; doi:10.1042/BCJ20220209)
Supplement: Supplementary Material 1 [file BCJ-479-1441-s1.pdf]

Supplementary Table 1: Additional SIM2 (NM_005069.6) gene variants found by clinical exome sequencing assayed in this study.

**Sex**	**Age (years)**	**Phenotype**	**Variants thought to explain phenotypes**	**Nucleotide (Amino Acid)** *Polyphen-2*	**Reads**	**gnomAD Database (allele frequency)**
F	5.8	Acute renal and liver dysfunction, enteropathy, heart disease, global developmental delay, mild hypotonia, dysmorphic features, short statue, failure to thrive, mild structural brain abnormality, and an abnormal N-glycan and transferrin test. Unusual movements possibly related to seizures associated with Streptococcus pneumonia bacteremia		**c.280G>A (p.V94M)** *probably damaging*	31/72	Not present
F	20.7	Postural orthostatic tachycardia syndrome (POTS), possible seizures, abnormal movements (random flinching), gastrointestinal symptoms, slight scoliosis and skin anomalies.		**c.322G>A (p.A108T)** *probably damaging*	46/85	0.00001063
M	66.0	Bilateral hearing loss, multifactorial gait difficulty, balance issues, weakness, peripheral neuropathy, cerebellar atrophy, short term memory loss, vitamin B12 deficiency and history of concussion.	Heterozygous c.35delG (p.G12fs) variant in the *GJB2* gene, a common pathogenic variant associated with autosomal recessive deafness (1). A novel hemizygous c.197C>A (p.A66D) variant of unknown significance (VUS) in the *BCAP31* gene. *BCAP31* variants cause deafness, dystonia, and cerebral hypomyelination (DDCH), an X-linked recessive disorder with phenotypes including lack of psychomotor development, dysmorphic facial features, deafness, dystonia and cerebral hypomyelination(2).	**c.515A>T (p.N172I)** *probably damaging*	21/53	0.000003977
F	7.9	Prematurity, delayed speech, refractory epilepsy, structural brain abnormalities (right frontal developmental venous anomaly), chronic otitis media, and persistent asthma.		**c.1146A>C (p.R382S)** *probably damaging*	83/184	0.000007964

Supplementary References

1. Kenneson A, Van Naarden Braun K, Boyle C. GJB2 (connexin 26) variants and nonsyndromic sensorineural hearing loss: A HuGE review. Genetics in Medicine. 2002;4(4):258-74.

2. Cacciagli P, Sutera-Sardo J, Borges-Correia A, Roux J-C, Dorboz I, Desvignes J-P, et al. Mutations in BCAP31 Cause a Severe X-Linked Phenotype with Deafness, Dystonia, and Central Hypomyelination and Disorganize the Golgi Apparatus. The American Journal of Human Genetics. 2013;93(3):579-86.
